# Cancer-associated fibroblasts promote cancer cell growth through a miR-7-RASSF2-PAR-4 axis in the tumor microenvironment

**DOI:** 10.18632/oncotarget.13609

**Published:** 2016-11-25

**Authors:** Zongze Shen, Xing Qin, Ming Yan, Rongrong Li, Gang Chen, Jianjun Zhang, Wantao Chen

**Affiliations:** ^1^ Department of Oral and Maxillofacial Head and Neck Oncology, Ninth People's Hospital, Shanghai Jiao Tong University School of Medicine, Shanghai 200011, China; ^2^ Shanghai Key Laboratory of Stomatology and Shanghai Research Institute of Stomatology, Shanghai 200011, China

**Keywords:** head and neck cancer, cancer microenvironment, cancer-associated fibroblasts, miR-7, RASSF2

## Abstract

Cancer-associated fibroblasts (CAFs), a major component of cancer stroma, play an important role in cancer progression but little is known about how CAFs affect tumorigenesis and development. MicroRNAs (miRNAs) are small non-coding RNAs that can negatively regulate target mRNA expression at post-transcriptional levels. In head and neck cancer (HNC), our analysis of miRNA arrays showed that miR-7, miR-196 and miR-335 were significantly up-regulated in CAFs when compared with their paired normal fibroblasts (NFs). FAP, α-SMA and FSP, specific markers of CAFs, were significantly expressed in CAFs. Functionally, exogenous expression of miR-7 in NFs induced a functional conversion of NFs into CAFs. In contrast, inhibition of miR-7 expression in CAFs could induce a functional conversion of CAFs into NFs. Our study demonstrated that overexpression of miR-7 in NFs significantly increased the migration activity and growth rates of cancer cells in co-culture experiments. Mechanistically, we confirmed that the RASSF2-PAR-4 axis was mainly responsible for miR-7 functions in CAFs using bioinformatics methods. Overexpression of miR-7 in CAFs led to down-regulation of RASSF2, which dramatically decreased the secretion of PAR-4 from CAFs and then enhanced the proliferation and migration of the co-cultured cancer cells. Thus, these results reveal that the inactivation of the RASSF2-PAR-4 axis controlled by miR-7 may be a novel strategy for gene therapy in HNCs.

## INTRODUCTION

Head and neck cancer (HNC), mainly consisting of squamous cell carcinoma, occurs in middle-aged to elderly adults and has a poor prognosis. Recently, the incidence of HNC increased in individuals younger than 45 years of age [1, 2]. Current treatments of HNCs include surgery, radiation and chemotherapy. However, a wide-range of operations causes dysfunction of the oral cavity, which deeply harms a patient's quality of life.

According to previous studies [3, 4], tumorigenesis is related to the surrounding environment, termed the cancer microenvironment. The cancer microenvironment is composed of many cells, such as stromal cells, fibroblasts and endotheliocytes. These cells promote the proliferation, invasion, migration and metastasis of cancer cells. In this microenvironment, cancer cells can establish cross-talk with stromal cells in autocrine or paracrine manners via chemokines, cytokines and inflammatory factors to change the signaling pathways and biological functions of cancer [5]. CAFs play an important role in the cancer microenvironment by establishing cross-talk with cancer cells. A published study has reported that cancer cell-secreted SDF-1 could be transported to CAFs, which in turn increased the invasion, migration and proliferation of cancer cells. In contrast, NFs inhibited these activities of cancer cells. However, it is still unknown how NFs transform into CAFs and whether the transformation could induce the malignant activity of cancer [6, 7].

MiRNAs are small non-coding RNAs that can negatively regulate target mRNA expression at post-transcriptional levels at the 3′UTR of target genes, which could result in alteration of mRNA expression and affect the biological function of cells [8]. In our study, we aimed to determine the key miRNAs that transform NFs into CAFs. Recent reports suggested that CAF-derived miR-31, miR-155 and miR-148 [9–11] could enable CAFs to significantly promote cancer proliferation, invasion and metastasis [12]. A study by Mitra [13] indicated that human ovarian cancer up-regulated miR-155 and down-regulated miR-214 and miR-31, which could reprogram normal fibroblasts into CAFs. Aprelikova's [14] study indicated that miR-148a was down-regulated in CAFs compared with NFs in endometrial cancer. MiR-148a regulated WNT10B and stimulated the activity of the Wnt signaling pathway, thus accelerating the tumor progression.

The present study indicated that miR-7 was significantly up-regulated in CAFs compared with NFs in HNCs by microRNA array analysis. We also predicted and confirmed that Ras association domain family member 2 (RASSF2) was a new miR-7 target gene using bioinformatics analysis and a dual luciferase reporter assay. RASSF2 is a pro-apoptotic effector in a variety of tumors [15], and a loss of RASSF2 expression enhanced the proliferation and migration of cancer cells. Prostate apoptosis response-4 (PAR-4) is a tumor suppressor that is secreted by normal cells. PAR-4 appears to induce cancer cell apoptosis by activating FAS and the TNF-TRAIL pathway [16]. The present study showed that PAR-4 was a direct binding partner of RASSF2. Low expression of RASSF2 impaired the ability of PAR-4 induced apoptosis in cells. We demonstrated that the inactivation of the RASSF2-PAR-4 axis mediated by activation of miR-7 may be a novel candidate for targeted therapy in HNCs.

## RESULTS

### Primary fibroblast culture and identification

**Figure 1:**
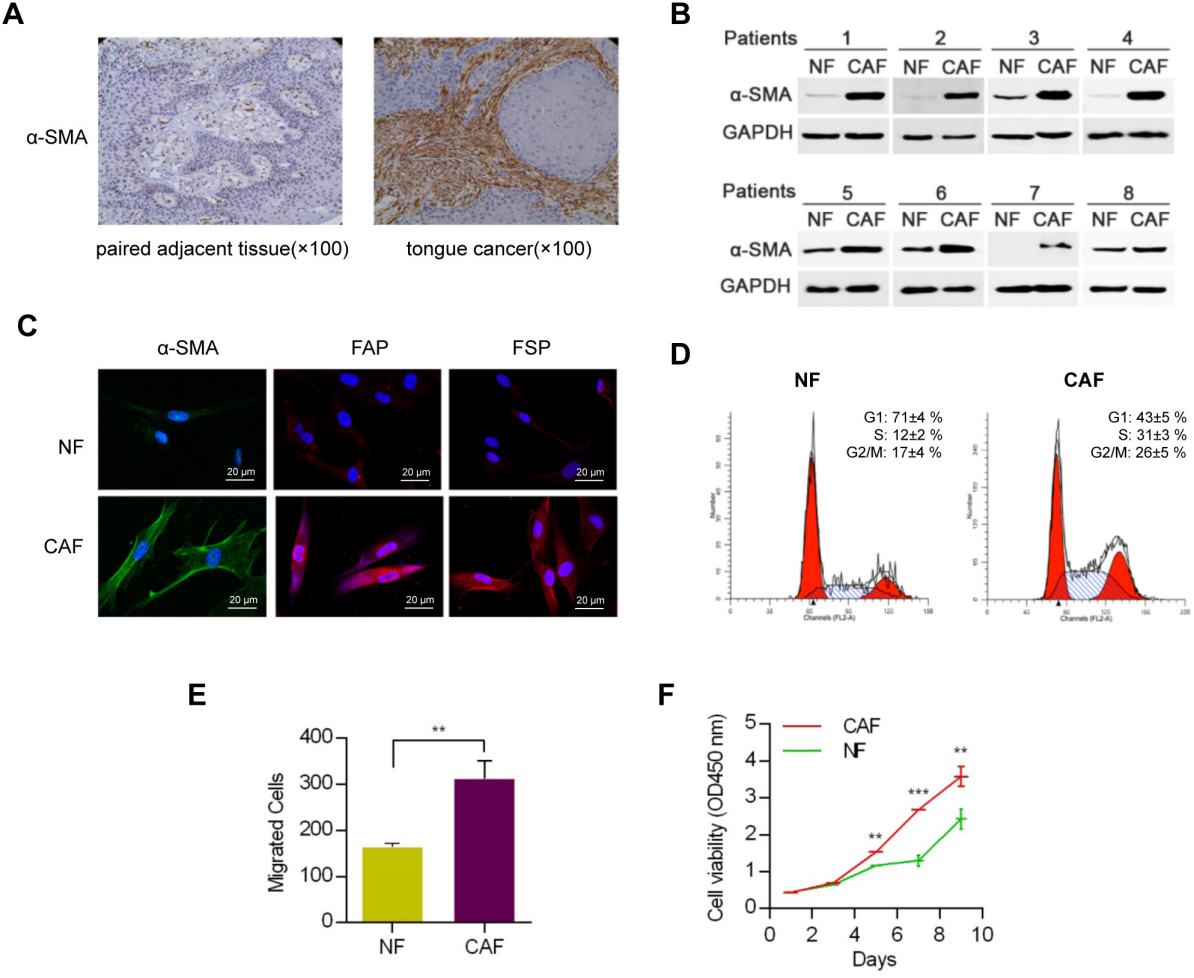
Identification and functional analysis of NFs and CAFs **A.** Images of immunohistochemical staining showed the distribution of α-SMA-expressing fibroblasts. **B.** Identification of NFs and CAFs using western blot analysis. **C.** FSP and FAP, specific biomarkers of CAFs, were proved to up-regulation in CAFs using immunofluorescence analysis. **D.** Cell cycle analysis of CAFs and NFs. **E.** The migratory ability of NFs and CAFs were determined by transwell migration assay. **F.** Proliferation analysis of CAFs and NFs. (** *p*<0.01; ***p<0.001.)

The location of CAFs and NFs in cancer tissues and paired adjacent normal tissues was identified by the immunostaining intensity of α-smooth muscle actin (α-SMA) (Figure 1A). The results showed that the α-SMA was seldom expressed in the paired adjacent normal tissues but was highly expressed in the CAFs, which surrounded the squamous cell carcinoma cell nests. According to recent reports [17], these fibroblasts with positive α-SMA expression were CAFs. Therefore, CAFs and NFs cells were cultured from cancer tissues and their paired adjacent normal samples by primary culture. The α-SMA expressions in the 8 established pairs of CAFs and NFs were tested by western blotting (Figure 1B), and the results revealed that CAFs expressed significantly high levels of α-SMA. Fibroblast activation protein (FAP) and fibroblast specific protein (FSP), specific biomarkers of CAFs, were observed by immunofluorescence and the same result was gained (Figure 1C). The results revealed that the fibroblasts obtain from the HNC tissues could be regarded as CAFs according to the previous study (Figure 1B and 1C).

### Characteristics of CAFs and NFs

To analyze the characteristics of the two types of fibroblasts, a series of experiments including migration and proliferation assays were performed for further study. CAFs or NFs cells were cultured to three generations before experiment. The cell cycle analyses showed that the ratio of S phase of CAFs was higher compared to NFs (Figure 1D). The transwell migration assay showed that CAFs had a more significant migration ability than that of NFs (Figure 1E). In addition, a proliferation test showed that CAFs grew faster than NFs (Figure 1F).

### MiRNA expression profile of NFs and CAFs

**Figure 2:**
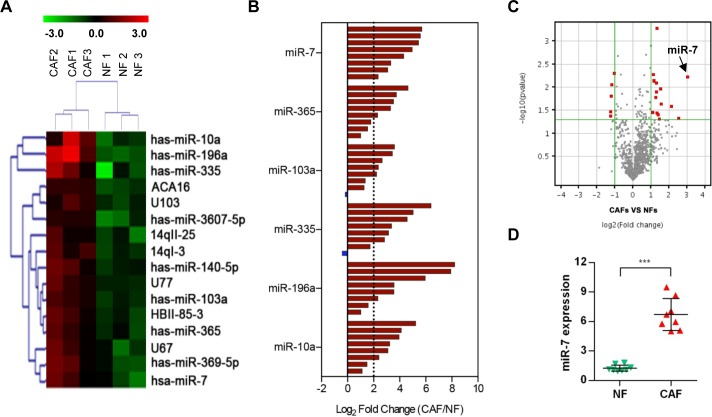
MicroRNA array analysis of CAFs **A.** Heat map of differentially expressed microRNA in CAFs compared with NFs. **B.** The expression levels of 6 miRNAs (miR-7, miR-365, miR-103a, miR-335, miR-196a and miR10a) in 8 pairs of NFs and CAFs. A log2-fold change more than 2 was regarded as significant up-regulation (dotted lines). **C.** A volcano plot showing the relationship between the P values and the magnitude of the differences in the expression values of the samples in different groups. **D.** A real-time PCR analysis was performed to validate the miR-7 expression in 8 paired samples. (***p<0.001.)

The miRNA expression levels were analyzed with a miRNA array in 3 pairs of NFs and CAFs, which were derived from the patients with HNC who underwent an operation. Among the 2006 miRNA capture probes, 16 probes (fold change ≥ 2 and *p*<0.05) were overexpressed in CAFs compared with their paired NFs. The microRNA array analyses confirmed that 9 miRNAs were highly expressed in CAFs. The 9 miRNAs were miR-7, miR-10a, miR-196a, miR-103a, miR-140-5p, miR-335, miR-369-5p, miR-365 and miR-3607-5p (Figure 2A).

To confirm the accuracy of microarray data, we selected the top 6 high-ranked miRNAs in CAFs and detected the expression changes of them in 8 pairs of NFs and CAFs (Figure 2B). A log2-fold change more than 2 was regarded as significant up-regulation (dotted lines). These data revealed that the miR-7, miR-365, miR-103a, miR-335, miR-196a and miR10a expression levels were increased in CAFs (Figure 2B): miR-7 was overexpressed in 100% (8 of 8) of the 8 cases of CAFs; miR-365 was overexpressed in 62.5% (5 of 8) of the 8 cases of CAFs; miR-103a was overexpressed in 62.5% (5 of 8) of the 8 cases of CAFs; miR-335 was overexpressed in 75% (6 of 8) of the 8 cases of CAFs; miR-196a was overexpressed in 75% (6 of 8) of the 8 cases of CAFs; and miR-10a was overexpressed in 75% (6 of 8) of the 8 cases of CAFs. Considering the consistency and stability of these high-ranked miRNAs (Figure 2B and 2C), miR-7 was more suitable to be regarded as a candidate gene for further study. The miR-7 expression level was verified in 8 additional paired CAFs and NFs by real-time PCR and the same result was obtained (Figure 2D). These data suggested that a set of miRNAs is aberrantly expressed in CAFs derived from HNC tissues and miR-7 might play an important role in the functional conversion of NFs into CAFs.

### Function of miR-7 in CAFs and NFs on HNC cells

**Figure 3:**
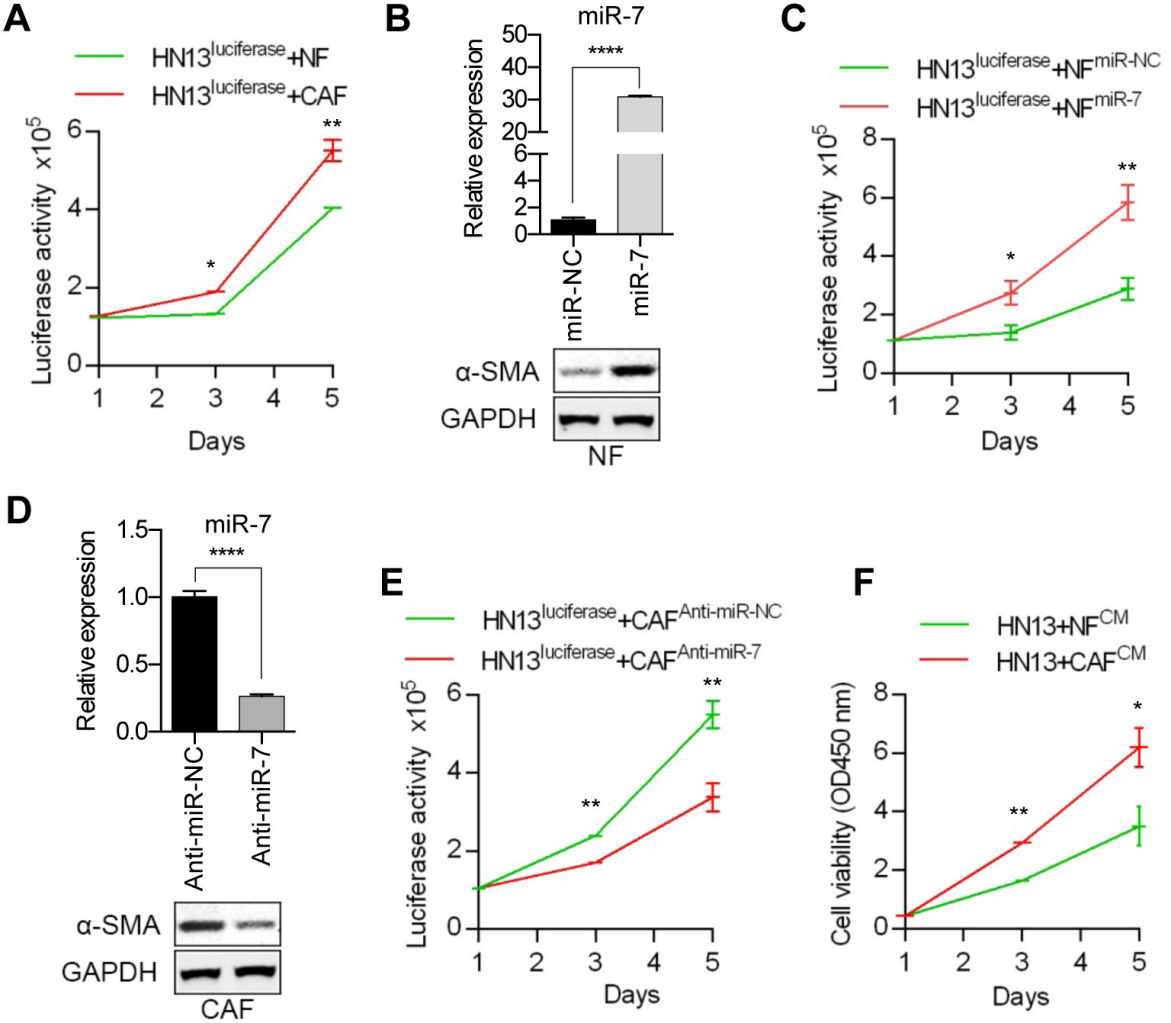
Functional analysis of miR-7 effects on the HNC cell proliferation **A.** Compared with NFs, CAFs could promote the proliferation of HN13 cells more efficiently. **B.** The results of real-time PCR showing miR-7 expression levels in NFs after transfection of miR-7 mimics and higher expression of α-SMA was detected in NFs^miR-7^ using Western blot analysis. **C.** Overespression of miR-7 in NFs could facilitate the growth of HN13 cells. **D.** The results of real-time PCR showing miR-7 expression levels in CAFs after treated with anti-miR-7 and decreased expression of α-SMA was detected in CAFs^Anti-miR-7^ using Western blot analysis. **E.** Silencing the expression of miR-7 in CAFs inhibited the proliferation of HN13 cells. **F.** CAF-derived CM exhibited a more proliferative effect on HN13 cells than that of NFs. (*p<0.05; ** *p*<0.01; ****p<0.0001.)

To detect the effect of the miR-7 expression levels in NFs or CAFs on the proliferation of cancer cells. The HNC cell line HN13 was first stably transfected with a lenti-luciferase vector and called HN13^luciferase^ and the cell number was quantified by measuring the luciferase activity of the co-culture system. Compared with NFs, CAFs could promote the proliferation of HN13^luciferase^ cells (Figure 3A). We found that exogenous miR-7 expression in NFs (Figure 3B) would up-regulate the expression of α-SMA and facilitate the proliferation of HN13^luciferase^ cells (Figure 3B and 3C). Meanwhile, silencing the miR-7 expression by anti-miR-7 in CAFs (Figure 3D) could partially down-regulate the α-SMA expression and decrease the growth rate of HN13^luciferase^ cells (Figure 3D and 3E). In addition, HN13^luciferase^ cells cultured with CAF-derived conditioned medium (CM) exhibited a more significant proliferative activity than that of NFs (Figure 3F). These data indicated that miR-7 might play a functional role in the conversion of NFs into CAFs to some extend and created a tumor-supportive microenvironment for the proliferation of HNC cells. What's more, we inferred that CAFs might affect the biological behaviors of HNC cells by some secreted molecular associated with miR-7.

### Identifying the targets of miR-7 in CAFs

**Figure 4:**
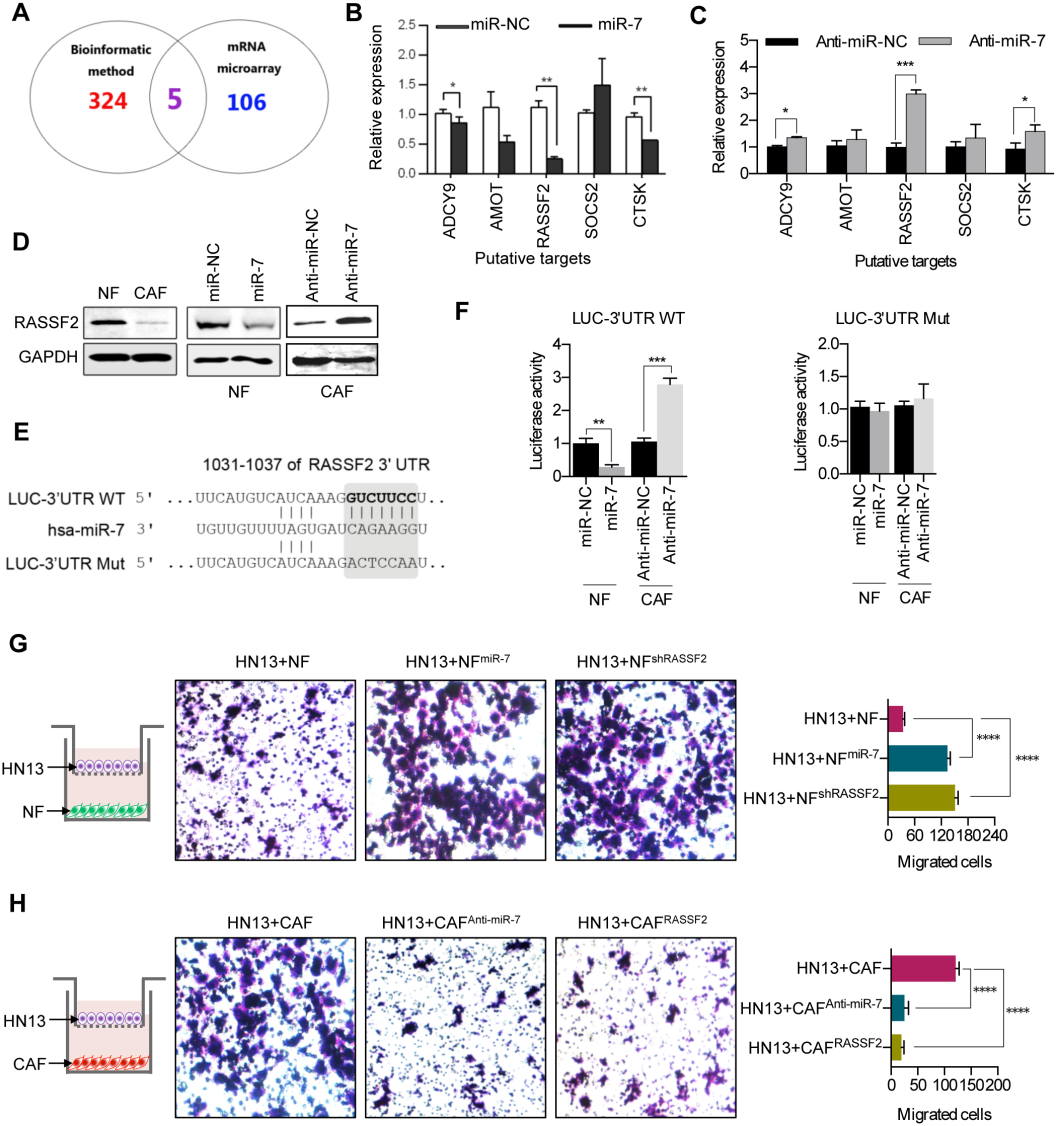
MiR-7 targets RASSF2 **A.** Venn diagrams of the predicted candidate target genes of miR-7 by bioinformatic method and mRNA microarray. **B, C.** The putative target genes were futher identified in NFs^miR-7^ and CAFs^Anti-miR-7^ using real-time PCR analysis. **D.** Western blot analysis of RASSF2 protein expression after altering miR-7 expression in NFs and CAFs. **E.** The schematic diagram of putative miR-7-binding sites in the 3′-UTR of RASSF2. **F.** Down-regulation in NFs^miR-7^ and up-regulation in CAFs^Anti-miR-7^ of the reporter gene with the wild-type region from RASSF2-WT was apparent, whereas no effect on the RASSF2-MUT was detected. **G, H.** Overexpression of miR-7 or knock-down of RASSF2 in NFs could accelerate migration of HN13 cells, whereas decreasing the miR-7 expression or up-regulation of RASSF2 in CAFs severely inhibited the migratory ability of HN13 cells. (*p<0.05; ** *p*<0.01; ***p<0.001; ****p<0.0001.)

MiRNAs suppress the expression of their target genes by binding to the specific sites of the 3′-untranslation region of mRNA to block the translational expression levels of target genes. The candidate target genes of miR-7 were first predicted using a bioinformatics method. To increase the accuracy of this prediction, target genes were predicted by at least three of 11 databases (TargetScan, MicroInspector, RNA22, miRanda, NBmiRTar, MirTarget2, miTarget, PicTar, PITA, RNAhybri and Diana). An intersection of the predicted genes and the down-regulated genes in a gene array (data not shown) of CAFs was assessed, and five candidate target genes were initially verified (Figure 4A). Real-time PCR assays were performed to observe the expressions of the five candidate target genes in NFs treated with miR-7 mimic and in CAFs transfected with anti-miR-7. The expression levels of RASSF2, ADCY9 and CTSK were significantly down-regulated in miR-7-treating NFs (Figure 4B), while up-regulations of the three genes were also observed in CAFs treated with anti-miR-7 (Figure 4C). Among the three genes, the expression of RASSF2 was observed to be remarkably changed (Figure 4B and 4C). These results revealed that the direct target gene for miR-7 was RASSF2. Western blot analysis showed that the RASSF2 protein expression was lower in CAFs compared to NFs (Figure 4D left panel). Exogenous overexpression of miR-7 decreased RASSF2 protein expression in NFs (Figure 4D middle panel). Blocking miR-7 expression using anti-miR-7 in CAFs increased RASSF2 expression (Figure 4D right panel). Meanwhile, there is an evolutionarily conserved binding site for miR-7 in the 3′UTR of RASSF2 gene (Figure 4E). Using 3′UTR luciferase reporter assays, we confirmed that RASSF2 was a direct target of miR-7 (Figure 4F). As showed in Figure 4F, miR-7 dramatically inhibited the luciferase activity in NFs and decreasing the expression of miR-7 in CAFs would obviously increase the luciferase activity. In contrast, the luciferase reporter mutated at miR-7 binding sites abolished the response to miR-7 (Figure 4F). Therefore, we confirmed that RASSF2 is a target gene of miR-7.

To determine if RASSF2 can mediate the biological function of miR-7, we performed a transwell assay to test the migratory activity of HN13 cells co-incubated with NF, NF^miR-7^, NF^shRASSF2^, CAF, CAF^anti-miR-7^ or CAFs^RASSF2^. The data showed that NF^miR-7^ and NF^shRASSF2^ induced the migration of HN13 cells at the same level (Figure 4G and 4H). In contrast, CAF^anti-miR-7^ and CAFs^RASSF2^ dramatically inhibited the migration of HN13 cells (Figure 4G and 4H).

### MiR-7-RASSF2-PAR-4 axis mediated the cross-talk of CAFs and cancer cells

**Figure 5:**
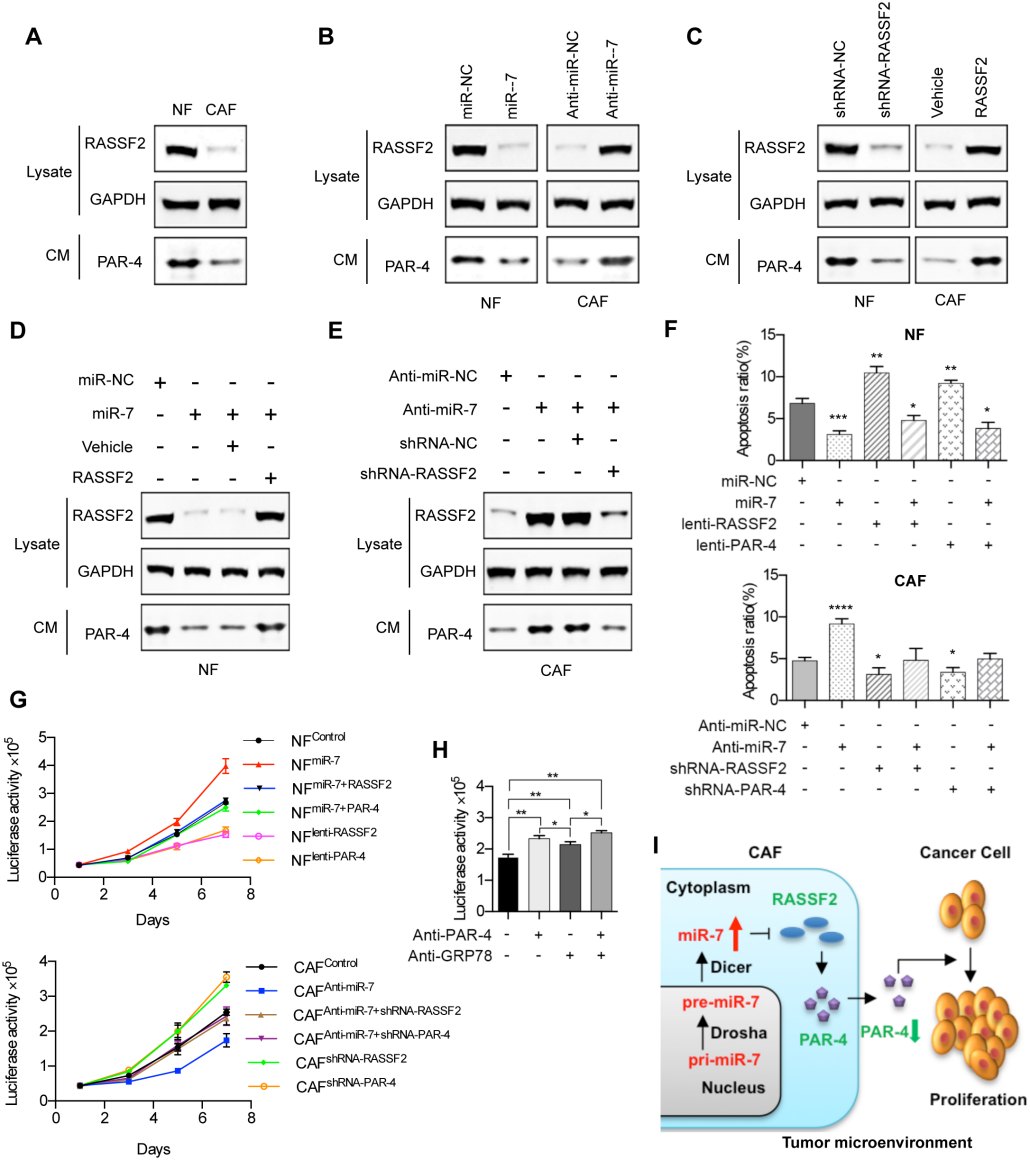
MiR-7-RASSF2-PAR-4 axis mediated the cross-talk of CAFs and cancer cells **A.** Western blot analysis of RASSF2 protein expression in NFs and CAFs, and the secreted PAR-4 expression in the CM. **B.** The protein expressions of RASSF2 and secreted PAR-4 were determined in miR-7-expressing NFs, or CAFs transfected with anti-miR-7 using western blot analysis. **C.** The protein expression levels of RASSF2 and secreted PAR-4 in RASSF2-expressing NFs, or RASSF2-silenced CAFs. **D.** The expression of PAR-4 was down-regulated by the overexpression of miR-7 and this was rescued by coexpression of RASSF2. **E.** The increased expression of PAR-4 in CAFs driven by anti-miR-7 could be reduced by siRNA for RASSF2. **F.** Exogenous RASSF2 or PAR-4 facilitated the apoptosis of NFs, whereas miR-7 could partly attenuate RASSF2- or PAR-4-induced apoptosis and vice versa in CAFs. **G.** Exogenous RASSF2 or PAR-4 decreased the growth rate of NFs, whereas miR-7 could partly attenuate RASSF2- or PAR-4-induced proliferative inhibition and vice versa in CAFs. **H.** The soluble PAR-4 acts on HNC cells by binding to the surface receptor GRP78. **I.** Flow diagram illustrating the cross-talk of CAFs and cancer cells mediated by the miR-7-RASSF2-PAR-4 axis. (*p<0.05; ** *p*<0.01; ***p<0.001; ****p<0.0001.)

A previous study [16] demonstrated that PAR-4 was a direct binding partner of RASSF2 and the lower expression of RASSF2 could impaire the PAR-4-driven apoptosis in tumor cells. Additionally, as PAR-4 is a secreted protein, we extracted the protein from the CM of CAFs and NFs to detect the protein level of PAR-4. The data showed that the PAR-4 protein was enriched in the conditional medium of NFs, but not in the CM of CAFs (Figure 5A).

To investigate if PAR-4 is a downstream signaling molecule for miR-7, we measured the PAR-4 expression level in the CM of CAFs and NFs when miR-7 expression was changed. The expression levels of RASSF2 and PAR-4 were decreased when NFs were treated with miR-7 mimics while an increased expression of RASSF2 and PAR-4 were observed when miR-7 was blocked in CAFs (Figure 5B).

To further identify if PAR-4 is a downstream signaling molecule for RASSF2, we detected the PAR-4 expression level in the CM of CAFs and NFs when RASSF2 expression was changed. The results revealed that silencing RASSF2 in NFs by shRNA could decrease the expression of PAR-4 while an increased expression of PAR-4 was observed when RASSF2 was overexpressed in CAFs (Figure 5C).

Evidence that RASSF2 is a key mediator for the cross-talk between miR-7 and PAR-4 came from the following set of observations: the expression of PAR-4 was down-regulated by the overexpression by miR-7 and this was rescued by coexpression of RASSF2 cDNA lacking the 3′UTR that restored RASSF2 protein to near endogenous levels (Figure 5D); in contrast, the increased expression of PAR-4 in CAFs driven by anti-miR-7 was reduced by siRNA for RASSF2 (Figure 5E).

RASSF2, a well-defined pro-apoptotic effector of K-Ras [15], was proved to be a direct target gene for miR-7 in HNC. Simultaneously, PAR-4, a direct binding partner of RASSF2, was defined as an essential gene for inducing apoptosis by activating FAS and the TNF-TRAIL pathway [16]. Since the expression of miR-7 was associated with the growth of HNC cells, we tried to verify whether miR-7 regulate apoptosis of HNC CAFs, or whether miR-7 regulated the apoptosis of CAFs trough the miR-7-RASSF2-PAR-4 axis. The results were in accordance with what have been mentioned above: overexpression of miR-7 in NFs could decrease the apoptosis of NFs, while anti-miR-7 could induce the apoptosis of CAFs; Furthermore, exogenous RASSF2 or PAR-4 drastically facilitated the apoptosis of NFs, whereas miR-7 could partly attenuate RASSF2- or PAR-4-induced apoptosis and vice versa in CAFs (Figure 5F). To well demonstrate the role of RASSF2-PAR-4 signaling axis in miRNA-7-associated CAF-driven cancer cell growth, we have designed a series of *in vitro* experiments. As shown in Figure 5G, overexpression of miR-7 in NFs could triger the proliferation of NFs, while anti-miR-7 could inhibit the growth of CAFs; Furthermore, exogenous RASSF2 or PAR-4 drastically decreased the growth rate of NFs, whereas miR-7 could partly attenuate RASSF2- or PAR-4-induced proliferative inhibition and vice versa in CAFs.

Recent reports further established that extracellular PAR-4 binded to cell surface receptor GRP78 via its SAC domain and activated the extrinsic apoptotic pathway [18, 19]. In this study, we proved that fibroblast-derived PAR-4 could inhibit the proliferation of HNC cells. However, it is not clear how PAR-4 plays its function in this process. As GRP78 is also an essential molecular chaperone and a master regulator of the unfolded protein response, blocking GRP78 by pharmaceutical or genetic methods would severely affect the cellular biological behaviors. Hence, we tried to block GRP78 by specific N-terminal-GRP78 antibody (NT-GRP78/N-20, Santa Cruz, sc-1050), which could specifically block the surface GRP78 as well as its inducible function [19]. As a result, we found that PAR-4-inducble proliferative inhibition of HNC cells was relieved by the specific N-terminal-GRP78 antibody. These data implied that the soluble PAR-4 acts on HNC cells by binding to the surface receptor GRP78 (Figure 5H).

In this study, we first identified the up-regulated expression of miR-7 in CAFs. Up-regulated miR-7 negatively controlled the target gene RASSF2 expression, which then decreased the secretion of PAR-4 into the cancer microenvironment. Previous studies demonstrated that PAR-4 was pro-apoptotic in cancer cells, so decreased secretion of PAR-4 from CAFs may be responsible for increased proliferative and migratory activity of cancer cells in the cancer microenvironment (Figure 5I).

## DISCUSSION

Cancer suppression or initiation and progression are reported to be associated with the surrounding microenvironment, which establishes cross-talk between cancer cells and matrix cells [20]. These cells affect tumor malignancy grade, such as metastasis, invasion and proliferation. The tumor microenvironment is highly heterogeneous and contains various cell types, including endothelial cells, immune cells and fibroblasts [21]. A recent study showed that a tumor-associated local extracellular matrix became altered to promote tumor progression and that the cancer-associated fibroblasts contributed to this response [22]. As our previous study indicated [23], the TGF-β3 inducing CAF-derived periostin in the microenvironment accelerated the proliferation and metastasis of the tumor cells in the primary site, and indirectly modulated a tumor-supportive microenvironment for the colonization initiation and metastasis of the tumor. In breast tumor, caveolin-1-negative CAFs were proved to provides a fertile soil for tumor cell growth [24]. Furthermore, evidences have indicated that in addition to promoting epithelial tumor growth, senescent fibroblasts can also promote carcinogenic initiation [25]. It was reported that the cell surface proteoglycan syndecan 1 was up-regulated in the malignant breast stromal fibroblasts, creating a favorable milieu for tumor cell growth [26]. What's more, ionizing radiation-mediated premature senescence and paracrine interactions with cancer cells colud enhance the expression of syndecan 1 in human breast stromal fibroblasts [26]. Interestingly, Maria et al. [27] reported that inhibiting the catabolic state in healthy cells could be a novel approach to improve current chemotherapy efficacies and possibly avoid the carcinogenic processes, after proteomic analysis of chemotherapy-induced CAFs. These data demonstrated that CAFs alone or the interactions with cancer cells played an important role in the tumor progression. In this study, we proved that CAFs could promote the proliferation and migration of HNC cells. Considering the complicated cross-talks between CAFs and cancer cells, further studies should be performed to reveal the mysterious nature of CAFs.

MiRNAs are non-coding RNAs that negatively regulate target gene expression and that participate in a variety of biological processes [28]. Therefore, we hypothesized that miRNAs played an important role in regulating specific genes, which could modulate the biological behaviors of cancer cells or create a favorable milieu for tumor initiation and progression. A recent study indicated that miR-335 was overexpressed in senescent CAFs and able to develop a senescence-associated secretory phenotype of CAFs that was believed to contribute to cancer progression [29]. In addition, there existed a miR-335-COX-2-PTEN axis which could regulated the secretory phenotype of senescent CAFs, thus creating a tumor-supportive microenvironment [29]. To screen out the dysregulated miRNAs in CAFs, we isolated the CAFs from HNC tissues and identified them by the recognized biomarkers of CAFs (α-SMA, FAP and FSP) using the methods of immunofluorescence, immunohistochemistry and western blot. The miRNA array showed that 9 miRNAs were up-regulated in CAFs. Specifically, miR-7 was the most significantly up-regulated miRNA. Some studies have indicated that miR-7 is a suppressive gene in tumors, but recent articles also argued that the miR-7 is an onco-miRNA in many cancers, including epithelial lung carcinoma, renal cell carcinoma of epithelial origin [30] and skin cancer, which could triger the proliferation, migration and initiation of tumor [31, 32]. In tumor microenvironment, little is known about the role of miR-7 in CAFs and the function of miR-7-expressing CAFs on tumor progression. Our data showed that the miR-7 was overexpressed in CAFs derived from HNC tissues and cellular functional tests revealed that miR-7 could promote the proliferative and migratory abilities of CAFs. Interestingly, miR-7, a potential key modulative gene for the functional conversion of NFs into CAFs, also displayed a promotive role in HNC cell proliferation and migration by modulating the function of CAFs. As described above, collective evidences have demonstrated that CAFs exhibit positive behaviors in tumor development. Hence, the overexpression of miR-7 in CAFs would only partially account for the active role of CAFs in tumor progression and further experiments should be performed to illustrate the mechanisms about the CAF-driven tumor development in future study.

As the previous study indicated, the Ras signaling pathway is always activated in many human tumors [33] and play an important role in many biological processes [34]. Ras proteins can induce cell division and oncogenesis by interacting with a wide range of effector proteins [35]. Via interactions with RASSF2, Ras proteins can mediate apoptosis and cell cycle arrest [36]. RASSF2, one of the six Ras-association domain family of genes, was found to located at 20p13 and methylation of the RASSF2 promoter was an early and a frequent event in a series of epithelial-derived tumours including breast cancer, colorectal cancer, non-small cell lung cancer, nasopharyngeal cancer et al. [37, 38]. Stable expression of RASSF2 suppressed cell growth and induced apoptosis, but a RASSF2 mutant lacking the Ras-associated domain was unable to interact with Ras and exhibited less pro-apoptotic activity [39]. These evidences revealed that RASSF2 was a novel K-Ras-specific effector and potential tumor suppressor. As noted above, many published studies have demonstrated that the RASSF2 gene is silenced by promoter methylation in numerous human cancers [37]. In the present study, we found that RASSF2 was a direct target gene for miR-7 and the decreased expression of RASSF2 in CAFs was associated with the up-regulation of miR-7. However, whether the methylation of RASSF2 promoter account for the down-regulation of RASSF2 is not clear. Thus, relevant experiemnts about the methylation of RASSF2 promoter in CAFs would be implemented in following study.

PAR-4, a pro-apoptotic protein with intracellular functions in the cytoplasm and nucleus, was reported to be spontaneously secreted by normal and cancer cells in culture [19]. Furthermore, endoplasmic reticulum (ER) stress could remarkerably increase cellular secretion of PAR-4 by a brefeldin A-sensitive pathway [19]. Previous studies [40, 41] have shown that PAR-4 is a tumor suppressor protein that can induce apoptosis in cancer cells. A recent study indicated that specific activation of p53 in NFs selectively induced the apoptosis of p53-deficient cancer cells which was mediated by p53-dependent secretion of the tumor suppressor PAR-4 in fibroblasts [42]. In addition, the elevated secretion of PAR-4 was induced by p53 activation, for p53 could suppress the expression of UACA to promote the expression of PAR-4 [42]. A previous study demonstrated that PAR-4 is a direct binding partner of RASSF2 [16]. Similarly, our study revealed that RASSF2 directly controlled the expression of PAR-4, thereafter inducing apoptosis in cancer cells. From our experiment, we found high levels of PAR-4 protein in the CM of NFs but not in the CM of CAFs, which indicated PAR-4 was secreted from stromal cells into the tumor microenvironment and then acted on the cancer cells. As displayed in this study, CAF-derived PAR-4 could promote the growth of HNC cells by binding to the surface receptor GRP78. However, it is still unclear whether the secreted PAR-4 would induce the apoptosis in tumor cells in the other way. Hence, further studies should be taken to investigate the functional role of the secreted PAR-4 on cell apoptosis.

As we all know, oral cancer is an important component of HNC. It is believed that oral cancer and lung cancer are malignant diseases which are closely associated with cigarette smoke. Exposure to cigarette smoke always leads to the dysregulation of some genes including oncogene and tumor suppressor genes, thus resulting in initiation of tumor [43]. The fragile histidine triad gene (FHIT), a tumor suppressor, was reported to be a common target of carcinogens, particularly cigarette smoke [44]. Evidences showed that FHIT loss could enhance the expression of a set of oxidative stress response genes after exposure to cigarette smoke extract, thereafter creating a survival advantage that promotes carcinogenesis [45]. Then the question becomes: whether the exposure to cigarette smoke extract would give rise to the dysregulation of some crutial genes (including miRNAs) in CAFs, or whether the CAFs derived from HNC tissues of smoking patient could create a more appropriate microenvironment to accelerate the carcinogenic process? Hence, further studies would be performed to investigate these clinicopathological problems in the future.

In this study, we first identified the up-regulated expression of miR-7 in CAFs. The overexpression of miR-7 negatively controlled the expression of target gene RASSF2, which then decreased the secretion of the PAR-4 protein into the cancer microenvironment. As a result, the decreased secretion of PAR-4 from CAFs were proved to be responsible for the promotion of HNC cell proliferation and migration. Therefore, these results reveal that the inactivation of the RASSF2-PAR-4 axis controlled by miR-7 may be a novel strategy for gene therapy in HNCs. Additionally, this mechanism is an important step toward regulating the tumor microenvironment, bringing us closer to understanding the potential of miRNAs in cancer treatment.

## MATERIALS AND METHODS

### Primary cell culture of CAFs and NFs

CAFs and NFs were derived from patients who underwent operations for HNCs at the Ninth People's Hospital, Shanghai Jiao Tong University School of Medicine. These fibroblasts were obtained by primary cell culture. First, tissues were washed with PBS containing 500 units/mL penicillin and 500 μg/mL streptomycin. Then, the tissues were cut into pieces and incubated with 0.25% trypsin-EDTA at 37°Cfor 10 mins in an incubator, centrifuged at 1000 rpm for 3 mins, transferred in to a culture dish and kept in a humidified 5% CO2 atmosphere at 37°C. Finally, all of the primary fibroblasts were cultured in Dulbecco's modified Eagle's medium (DMEM; GIBCO-BRL, BOSTON, MA, USA) supplemented with 10%fetal bovine serum (GIBCO-BRL, USA), penicillin (100 units/ml), and streptomycin (100 μg/ml) at 37°C in a humidified 5% CO2 atmosphere. A Cell-counting kit (CCK)-8 (Dojindo, Japan) was used to test the proliferation of CAFs and NFs according to the manufacturer's protocol. The CAFs and NFs were passaged each week. The second generation primary cells were purified by a differential adhesion method, and these cells were designated as the third generation. After the third generation, CAFs or NFs were used in cell experiments. The remainder of the CAFs and NFs were incubated using the above conditions. The CAFs and NFs grew well after 10 passages. We always used CAFs and NFs in experiments at 3-10 generations. The primary cell lines were assessed by western bolt, immunofluorescence and immunohistochemistry for specific biomarkers of CAFs (α-SMA, FSP and FAP).

### Head and neck cancer cell culture

HN13 cells were kindly provided by the Dental School of the University of Maryland and were cultured in DMEM (GIBCO-BRL, USA) medium containing5% fetal bovine serum, penicillin (100 units/ml) and streptomycin (100 μg/ml) [46].

### Western blotting

To obtain cell proteins, cells were washed in PBS three times and lysed by SDS in 4°C for 10 mins. The protein concentration was tested using a BCA Protein assay kit (Thermo, USA) and analyzed by microplate reader (TECAN, USA). The protein samples were electrophoresed through polyacrylamide gels and transferred ontoa 0.44 mm PVDF membrane (Millipore, USA). After blocking with 5% low-fat milk, the membrane was probed with mouse polyclonal antibody α-SMA (1:600; abcam, USA), GAPDH (1:8000; Kang Cheng, China) and incubated with rabbit polyclonal FSP and FAP (1:500; Abcam, USA) at 4°C overnight. After 12 hours, a secondary anti-mouse antibody for α-SMA and anti-rabbit antibody for FSP and FAP was applied. GAPDH was used throughout as a loading control. The membrane was then scanned using the Odyssey infrared imaging system (LI-COR, Lincoln, NE) at an 800 channel wave length and analyzed with Odyssey software.

### Immunofluorescence and immunohistochemistry

In the immunofluorescence experiment, we seeded 5×10^3^ CAFs or NFs onto a coverslip in 24-well plates, and the 24-well plates were washed with PBS and then immobilized for 20 mins at 4°C with paraformaldehyde, incubated in 1% Triton for 20 mins, and then 5% low fat milk to block the antigens for 20 mins, probed with a primary mouse polyclonal α-SMA antibody (1:50; Abcam, USA) and rabbit polyclonal FSP and FAP antibodies (1:60; Abcam, USA), and incubated at 4°C overnight. After 24 hours, the coverslips were stained with fluorescent secondary antibodies to observe the specific biomarkers. Finally, a confocal microscope (Leica, Germany) was used to observe the biomarker expression.

For immunohistochemistry, the cancer tissue samples and tumor adjacent tissue samples were collected from patients who underwent operations for HNC in the Ninth People's Hospital of the Shanghai Jiao Tong University School of Medicine. Briefly, tissue sections were deparaffinized in xylene, rehydrated in graded ethanol, treated with Tris-EDTA buffer for antigen retrieval, and quenched in hydrogen peroxide. The tissue sections were blocked with 2.5% normal serum. We probed with mouse polyclonal α-SMA (1:50; abcam, USA) on both groups of samples at 4°C overnight. After 24 hours, the slides were washed 3 times with PBS, incubated with secondary antibody and stained with diaminobenzidine (DAB) for 30 mins. Finally, α-SMA was observed according to the DAB coloration.

### Cell cycle analysis

For flow cytometry analysis, CAFs and NFs were trypsinized and fixed in 70% ice-cold ethanol overnight. The cells were then incubated in a 0.5-ml propidium iodide solution (BD, San Jose, CA, USA) at 50 mg/ml in PBS plus 25 mg/ml RNase in flow tubes for 15 mins at 37°C and were measured by flow cytometry (Thermo Fisher, USA).

### MiRNA array analysis

The miRNAs expression profiling of three paired CAFs and NFs was performed using a miRCURY LNA Array (v.14.0) system (KANGCHEN Bio-Tech, Shanghai, China) as described previously [47], Briefly, total RNA was extracted using TRIzol reagent (Invitrogen, shanghai, China) and miRNA was extracted using a kit specifically designed to capture these low molecular weight species (Exiqon A/S, Vedbaek, Denmark), according to the manufacturer's instructions. A NanoDrop 1000 UV-Vis Spectrophotometer (Nanodrop Technologies, Wilmington, USA) was used to measure the quantity of isolated RNA. The resulting RNA samples were labeled with a miRCURY Hy3/Hy5 Power labeling kit (Exiqon A/S, Vedbaek, Denmark) and hybridized on an miRCURY LNA Array (Exiqon A/S). After washing, the microarrays were scanned with an Axon GenePix 4000B microarray scanner (Axon Instruments Inc, Union City, CA, USA) and analyzed using Pro 6.0 software (Molecular Devices, LLC, USA). The miRNAs, reported intensities ≥30, were sent for further analysis. Normalization was carried out by transforming the expression of each gene into having a mean of 0 and a standard deviation (SD) of 1 and the statistical analysis was then performed. The miRNA data were analyzed as the ratio of CAFs/NFs cell signals. The presence of differentially expressed miRNAs was determined using volcano plot filtering and statistical significance was accepted for changes >2-fold. Finally, hierarchical clustering was performed to demonstrate distin- guishable miRNA expression profiling among the samples. The intensity of the green signal was calculated after background subtraction, and replicated spots on the same slide were averaged to obtain the median intensity.

### Real-time PCR assay

Total RNA was isolated from cells by TRIzol extraction (Invitrogen, shanghai, China) on ice in an RNA-free environment and then subjected to reverse transcription using the miRcute miRNA first-stand cDNA Kit (TIANGEN, Shanghai, China), according to the manufacturer's protocol. The miRNA analysis used poly (A) polymerase to add a poly (A) tail to total RNA before reverse transcription. Real-time PCR was performed under the following conditions: 1 cycle for degeneration 95°C/5 mins, 40 cycles for annealing 60°C/30 secs and extending 72°C/30 secs. The miRNA expressions were analyzed using SYBR Green methods and a miRcute miRNA qPCR kit according to the manufacturer's protocol (TIANGEN, Shanghai, China). The following primers were used to amplify human miR-7. The miR-7 forward primer was 5′-TGGAAGACTAGTGATTTTGTTGT-3′. The expression of targeted candidate mRNAs was tested using an mRNA qPCR kit (Premix Ex Taq, TaKaRa, China). The following primers were used to amplify human ADCY9, AMOT, RASSF2, SOCS2, CTSK genes. All of the primers were designed by primer 5.0 software. The primer sequences used were as follows: ADCY9 forward was 5′-CCGGCAAAGTTCACATTTCT-3′ and ADCY9 reverse was 5′-CAAACCTTTCA ACTGGTCAGC-3′. AMOT forward was 5′-TTAC CGTCTCTCCCAACCTG-3′ and reverse was 5′-ATAGGCTTCTCCTGGCTGCT-3′. The RASSF2 forward primer was 5′-TGAGGCAAGAGGATTTC TGG-3′ and the reverse primer was 5′-TCTG GTGAGGGAAGAGAGGA-3′. The SOCS2 forward primer was 5′-TGCCTTGCCTTCTTAGGTTC-3′ and the reverse primer was 5′-GGTTCCTTCCCACTTCTTCA-3′. The CTSK forward primer was 5′-GGCTCAAG GTTCTGCTGCTA-3′ and the reverse primer was 5′-TGCTTCCTGTGGGTCTTCTT-3′. The GAPDH forward primer 5′-CCTCTGACTTCAACAGCGAC-3′ and the reverse primer was 5′-TCCTCTTGTGC TCTTGCTGG-3′. The above experiments were performed according to the manufacturer's protocol for detecting mature miRNAs/mRNA, and U6/GAPDH was used as a standard for normalization. Real-time PCR reactions were amplified in a tube volume of 20 μl for 40 cycles using an Applied Biosystems 7300 real-time PCR system. All of the reactions were performed in triplicate.

### Migration assay

We plated 5×10^4^ NFs/CAFs into 24-well plates in 5% serum DMEM under the surface and 2×10^4^ HN13 cells in serum-free DMEM were plated on the upper surface of a transwell (Millipore, USA). Then, the cells were incubated for 24 hours in a humidified atmosphere containing 5% CO_2_ at 37°C. The cells that traversed through the membrane were fixed by methanol and stained with crystal violet. Images of four randomly selected fields were captured, and the cells were counted.

### Co-culture of HNC cells with CAFs or NFs

We used puromycin to screen HN13, which stably expressed firefly luciferase. In total, 3×10^3^ CAFs or NFs were co-cultured with luciferase-expressing HN13 cells for 5 days. We used a luciferase reporter (Firefly Luciferase Reporter Gene Assay Kit, Shanghai, China) to detect the luciferase activity which could reflect the proliferation of HN13 cells. Briefly, the cells were washed 3 times with PBS, treated with 250 μl cell lysis buffer, vibrated the cells for 15 mins at room temperature and centrifuged at 1200 rpm for 5 mins at 4°C. Finally, 50 μl of the above supernatant was mixed with 50 μl fluorescent substrate reagent and the luciferase activity was measured using using a Luciferase Assay System (Promega Corporation, USA).

### Construction of the RASSF2 luciferase reporter gene vectors

To obtain the full 3′UTR sequence of RASSF2, a template of cDNA of NFs was used for PCR. The 3′ UTR region of RASSF2 was cloned into the Xbal region of the pMD18T vector. Agarose gel electrophoresis after PCR amplification confirmed the products, and the amplification products were used in a restriction enzyme-Xbal digestion at 37°C overnight. The amplification products were cloned into the Xbal site of pGL3 control.

### Statistical analysis

The two variables of the microarray and qRT-PCR data were analyzed using the two-tailed Student's *t*-test. All of the data were expressed as the mean ± standard deviation. A *P* value <0.01 was considered statistically significant.

## ABBREVIATIONS

CAFs: Cancer-associated fibroblasts
miRNAs: microRNAs
HNC: head and neck cancer
NFs: normal fibroblasts
RASSF2: Ras association domain family member 2
PAR-4: prostate apoptosis response-4
CM: conditioned medium
ER: endoplasmic reticulum
FHIT: fragile histidine triad gene
α-SMA: α smooth muscle actin
FAP: fibroblast activation protein
FSP: fibroblast specific protein.
